# The Impact of Type 2 Diabetes Mellitus on the Severity of Alzheimer’s Disease in Hispanic Populations in the Rio Grande Valley: A Pilot Retrospective Chart Review

**DOI:** 10.7759/cureus.86086

**Published:** 2025-06-15

**Authors:** Maria Camila Gonzalez Tovar, Daniel Salinas, Kelsey Baker

**Affiliations:** 1 Neurology, University of Texas Rio Grande Valley School of Medicine, Edinburg, USA; 2 Neurology, University of Texas Rio Grande Valley School of Medicine, Harlingen, USA; 3 Neuroscience, University of Texas Rio Grande Valley School of Medicine, Edinburg, USA

**Keywords:** alzheimer’s disease, clinical assessment, diabetes mellitus, hispanic, latino, severity

## Abstract

Background: Alzheimer’s disease (AD) is becoming more prevalent worldwide, leading to a growing demand to understand the impact of risk factors on disease progression. Notably, type 2 diabetes mellitus (DM) has emerged as a significant risk factor for the development of AD, given the pathophysiological role of insulin resistance in cognitive impairment. In particular, the impact of type 2 DM on AD development and severity may be heightened in Hispanic populations due to the high prevalence of both conditions in this community. Here, we sought to understand the role of type 2 DM in AD severity in a Hispanic population from the Rio Grande Valley (RGV), a community wherein 91% of the population identifies as Hispanic.

Methods: We conducted a pilot retrospective chart review from January 2018 to March 2024 at UTHealth RGV for patients with AD who self-identified as Hispanic, Mexican, or Latino. Medical charts were evaluated for a diagnosis of type 2 DM, and demographics were recorded. We categorized the included charts into two groups: AD with DM and AD without DM. For all charts, the Global Deterioration Scale (GDS) score was manually determined from the electronic medical record. T-tests were used to evaluate differences between the two groups.

Results: Forty patients met the inclusion criteria for our study. Eleven medical charts were excluded (72.5% analysis rate) due to insufficient documentation for GDS scoring. No significant differences were found in baseline demographics between the AD with DM and AD without DM groups. GDS scores were not statistically different between groups (p = 0.152), although a medium effect size was observed (d = 0.52, 95% CI (-0.18, 1.23)), with higher GDS scores in the AD with DM group.

Conclusion: Our findings suggest a trend toward more severe AD in Hispanic patients with type 2 DM. Based on these results, we recommend the development of standardized assessment tools for AD, particularly for use in small community clinics, to improve the evaluation of disease progression. Improved clinical documentation and assessment may help identify risk factors that allow for earlier diagnosis or preventive interventions.

## Introduction

Alzheimer’s disease (AD) is the most common neurodegenerative disease, affecting roughly 50 million people worldwide, and is predicted to triple by 2050 [1]. The pathological diagnosis of AD requires the presence of amyloid β and phosphorylated tau proteins, while clinical diagnosis typically relies on cognitive criteria, such as performance on a neurological exam or the Montreal Cognitive Assessment (MoCA). Numerous studies have suggested that phosphorylated proteins contribute to AD’s varying stages of disease progression, ranging from cognitively normal to mildly cognitively impaired or dementia [2]. Despite the role of underlying pathology in clinical symptoms, it is well documented that AD is a multifactorial disease in which genetics, environment, and lifestyle also play pivotal roles in its development and progression to varying degrees [1,3]. In addition, comorbidities have been suggested to influence the diagnosis and severity of AD [1,3].

One comorbidity that has shown a growing association with AD is type 2 diabetes mellitus (DM). Notably, it has been shown that patients with type 2 DM have a 65% increased risk of developing AD [4]. Type 2 DM has also been demonstrated to lead to cognitive impairment and is considered one of the most prominent causes of cognitive decline in the elderly [2,3]. The link between AD and DM has been attributed to insulin resistance. Specifically, insulin resistance can result in hyperphosphorylation of tau protein and a buildup of amyloid β proteins in the brain, increasing the formation of senile plaques [2]. Therefore, DM may not only be a risk factor for the development of AD but also a contributing factor to its progression. This link has led some researchers to suggest that AD should be classified as “type 3 diabetes” or “brain diabetes” [4,5], given the role of insulin resistance in AD pathophysiology. However, a deeper understanding of AD pathogenesis is likely required before definitively linking AD to diabetes and insulin resistance.

The link between AD and type 2 DM holds particular importance in populations that commonly face socioeconomic disparities, such as Black and Latino communities. Latino/Hispanic individuals remain one of the fastest-growing populations in the United States [6]. Within this population, individuals are 1.5 times more likely to develop AD than non-Hispanic White individuals [6]. This is further compounded by data showing that type 2 DM rates are 80% higher in Hispanic adults and fivefold in Hispanic children compared to their non-Hispanic White counterparts in the United States [7]. In the Rio Grande Valley (RGV), a border region in the southern tip of Texas where 91.5% of the population identifies as Hispanic, one in three individuals has type 2 DM [8]. Therefore, evaluating the link between type 2 DM and AD, particularly in Hispanic populations, is critical. Such information may provide an opportunity for early detection and prevention.

The goal of this study was to evaluate the association between the presence of type 2 DM and AD severity. Specifically, we conducted a pilot retrospective chart review of all Hispanic patients with AD who visited our community clinic (UT Health RGV) over a five-year period. For all identified patients, we assessed AD severity using the Global Deterioration Scale (GDS), based on physician documentation. We hypothesized that Hispanic patients with both AD and DM would have significantly higher GDS scores compared to those without DM.

This article was previously presented as a poster at the University of Texas Rio Grande Valley (UTRGV) School of Medicine Research Colloquium on September 13, 2024.

## Materials and methods

Study design and medical chart inclusion

A retrospective chart review was conducted of UT Health RGV medical records from January 1, 2018, to March 1, 2024. We chose to use a retrospective design given its high external validity and increased statistical power. Inclusion criteria for the study were (1) patients with a coded diagnosis of AD (ICD-10 G30) and (2) individuals who self-identified as Hispanic, Latino, or Mexican. Disease diagnosis codes were assigned in both inpatient and outpatient settings (a total of 14 clinics) by neurologists, internists, or primary care providers. Information on how the diagnosis was made was not available, as each clinic followed its own standard protocol. For all included charts, we recorded type 2 DM status (ICD-10 code E11), hypertension status, and demographic information such as age, sex, and body mass index (BMI). Charts were excluded if they lacked data to determine a GDS rating.

The UTRGV Institutional Review Board (IRB) reviewed and approved all aspects of the study. A waiver of consent was granted by the IRB.

Evaluation of medical charts

Each included chart was manually reviewed, and a GDS score was assigned [9]. The GDS is a clinical scale that categorizes the stages of cognitive decline in individuals with neurodegenerative diseases into seven levels. Stages 1 through 3 are defined as pre-dementia, while stages 4 through 7 indicate various levels of dementia. The GDS is useful in identifying where a patient falls in the disease progression. All GDS scoring in this study was conducted by MCGT.

A GDS score of level 1 indicates no cognitive decline, and individuals in this category typically do not report any memory deficits.

Level 2 represents very mild cognitive decline, where individuals may subjectively report forgetting where familiar objects are placed or struggling to remember names.

Level 3 (mild cognitive decline) manifests as getting lost when traveling to unfamiliar places, coworkers noticing a decline in performance, close family observing word- and name-finding difficulties, retaining little of what is read, forgetting the names of recently met people, misplacing valuable objects, and having difficulty concentrating.

At Level 4, which corresponds to moderate cognitive decline, the patient shows decreased knowledge of current or recent events, memory deficits related to personal history, reduced concentration on serial subtraction tasks, and a decreased ability to manage finances or travel independently. The patient is also unable to perform complex tasks.

At Level 5, or moderately severe cognitive decline, the patient requires some assistance. They are unable to recall major relevant aspects of their life (e.g., their address or the names of their children), may be disoriented to time and/or place, and find it difficult to count backward by fours. However, they do not yet require help with toileting or eating.

At Level 6, representing severe cognitive decline, the patient may forget the name of their spouse, is unaware of recent events and surroundings, and has difficulty counting to 10. They require assistance with activities of daily living and exhibit personality and emotional changes.

At Level 7, which signifies very severe cognitive decline, all verbal abilities are lost, the patient is incontinent, and complete assistance is needed for toileting and feeding. Psychomotor skills are lost, and generalized rigidity is observed.

In this study, GDS scores were determined based on physician documentation related to patients’ activities of daily living and observed behavioral changes.

Statistical analysis

All statistical analyses were performed using R (R Foundation for Statistical Computing, Vienna, Austria) [10]. We used Fisher’s exact test to examine whether DM status influenced the likelihood of obtaining a GDS score, as the categorical nature of both variables and the small sample size violated assumptions required for the chi-square test. Once GDS scoring was completed, the data were divided into two groups: AD patients with DM and AD patients without DM. To compare mean differences in demographics, comorbidities, and GDS scores between the two groups, we used Welch’s two-sample t-test. This test was selected because it accommodates unequal variances and differing sample sizes. A p-value less than 0.05 was considered statistically significant.

## Results

Patient characteristics

**Figure 1:**
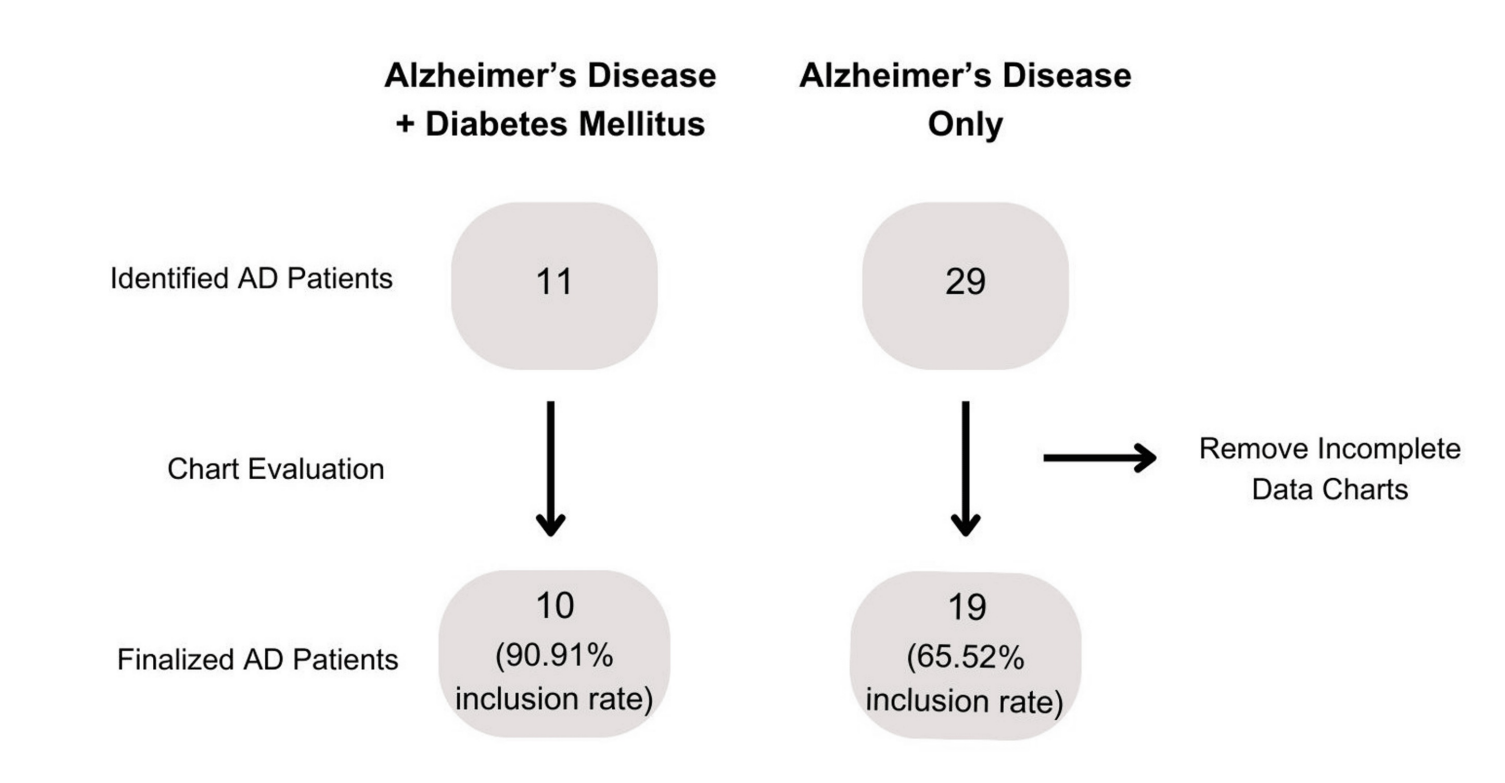
Study design and evaluation process A total of 40 patients were identified with AD in the study. All patient charts were reviewed to determine whether an AD severity score could be assigned. The inclusion rate for each group is shown at the bottom of the figure (90.91% for AD + DM and 65.52% for AD only). The ability to obtain a graded evaluation score in our dataset was not related to diabetes status (OR = 5.09, 95% CI [0.57, 250.54], p = 0.233). AD: Alzheimer’s disease; DM: diabetes mellitus.

A total of 40 patients with AD met the inclusion criteria and were included in the study. During chart review, 11 patients (10 without diabetes and 1 with diabetes) were excluded due to a lack of physician documentation required for assigning a GDS score. Specifically, most charts that were removed from analysis lacked documentation regarding the patient’s ability to perform activities of daily living and/or behavioral changes. However, our ability to obtain a graded evaluation score in our dataset was not related to diabetes status (OR 5.09, 95% CI (0.57, 250.54), p = 0.233). Therefore, 65.52% (n = 19) and 90.91% (n = 10) of identified AD patients without and with diabetes, respectively, were able to be evaluated in this study (Figure 1).

**Figure 2:**
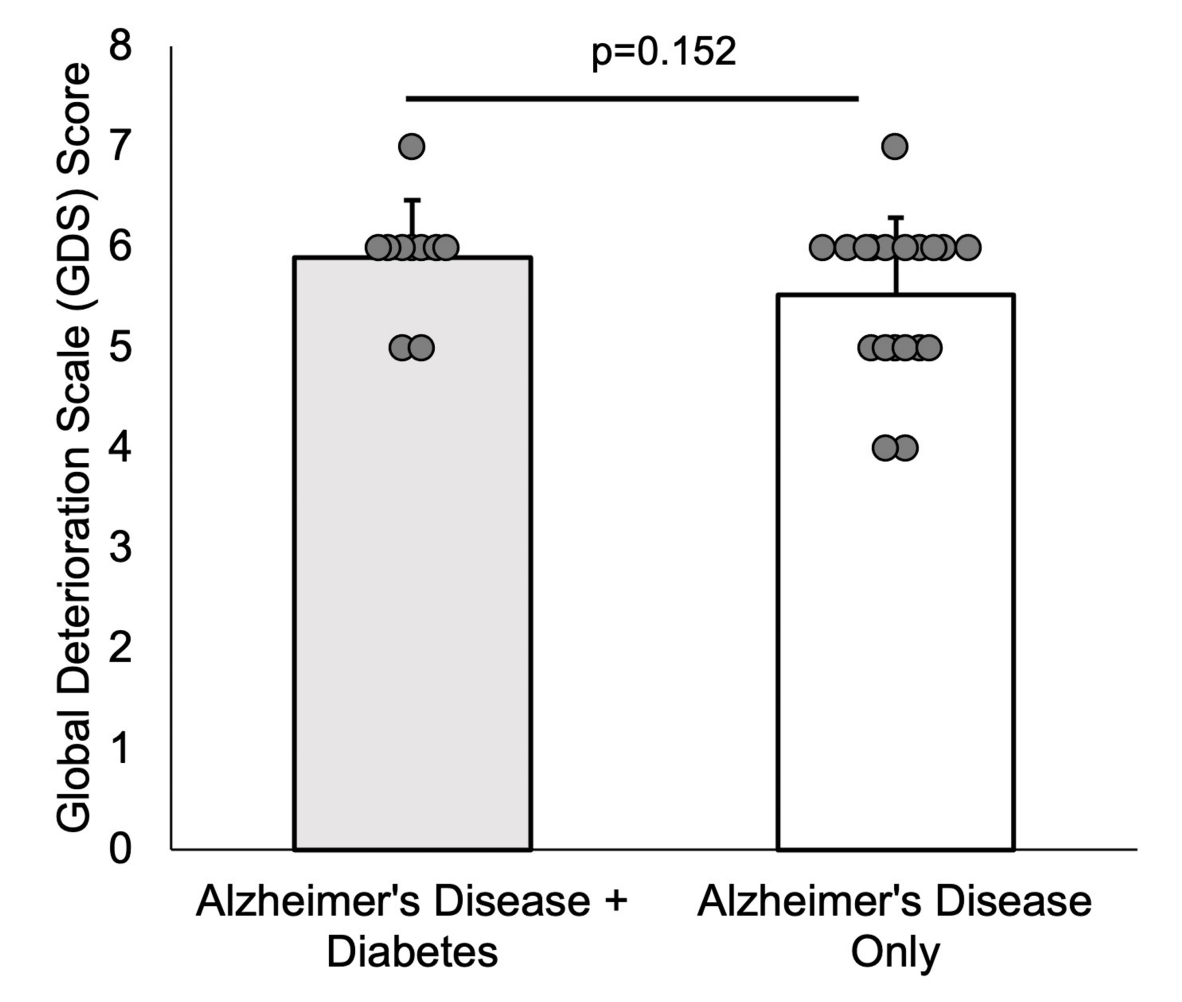
Global Deterioration Scale (GDS) score between groups The GDS score between Alzheimer’s disease patients with (gray bar) and without diabetes mellitus (white bar) demonstrated no significant difference between groups (p = 0.152).

We found that the baseline characteristics of the twenty-nine analyzed patients showed no significant differences between groups (Table 1). Next, we evaluated whether GDS scores differed between groups. There was no significant difference in GDS scores for individuals with AD without DM (M = 5.53, SD = 0.77) compared to those with AD and DM (M = 5.90, SD = 0.57) (t(23.79) = -1.48, p = 0.152; Figure 2). However, a medium effect size was observed (d = 0.52, 95% CI (-0.18, 1.23)), with higher graded evaluation scores for individuals with AD and DM. 

**Table 1:** General characteristics of the participants divided by the presence of diabetes mellitus Data are presented as mean (SD). GDS: Global Deterioration Scale; BMI: body mass index.

Variables	Alzheimer's Disease With Diabetes	Alzheimer's Disease Without Diabetes
Age (years)	85.1 (9.55)	81.37 (8.08)
BMI	26.26 (6.5)	24.35 (5.91)
GDS score	5.9 (0.57)	5.53 (0.77)
Hypertension diagnosis, n (%)	9 (90%)	15 (78.9%)
Sex		
Male (n)	3	10
Female (n)	7	9

## Discussion

We sought to determine whether the presentation of type 2 DM in AD would lead to a more severe disease presentation in a Hispanic population. Prior studies have shown that DM leads to cognitive impairment and, due to its pathophysiology, can be linked as a contributing factor to the progression of AD [2,3]. Furthermore, results have indicated that Hispanic populations may be more predisposed to develop what is being termed “brain diabetes,” or AD that is linked with insulin resistance [4,5]. However, we found that AD disease severity, as assessed by the GDS, was not significant between groups (p = 0.152), although a medium effect size (d = 0.53) was observed. This effect size suggests a potentially meaningful clinical difference that did not reach statistical significance due to the limited sample size. These findings indicate that additional data are needed to more accurately estimate the effect size and assess potential clinical relevance, while also offering insight into the direction these clinical tools may take in guiding future diagnostic or treatment strategies.

When considering what could have contributed to a nonsignificant result, we hypothesized that a few factors may have influenced our findings. First, since we collected data across 14 clinical sites as part of this pilot study, we were unable to ascertain the severity of DM in our analyzed patients. Thus, it could be that DM was well controlled in our sample, and this precluded significant findings. In addition, we cannot discount that our study population, Hispanic individuals, may have an unknown tolerance that prevented the comorbid diagnosis of DM from impacting the severity of AD. For example, DM has been found to be neuroprotective in some neurological conditions [11,12]. Future studies should consider evaluating DM management and its influence on the severity of AD in Hispanic populations. It is also possible that, since our data were collected in the RGV, a border town between the United States and Mexico, many to most of the population that we examined may have been bilingual to varying degrees. Bilingualism has been found to be a known protective factor against AD, which could have offset the effects of DM [13-15]. However, we believe this may have had a limited role in our current findings, since both our groups had similar exposure to social environments and environmental factors.

Even though we obtained forty patients available in the RGV after the inclusion criteria were met, only twenty-nine patients were evaluated. Specifically, only 65.52% of the available sample size of Alzheimer patients without diabetes (n = 19) were utilized, whereas 90.91% of the sample size of Alzheimer patients with diabetes (n = 10) were used (Figure 1). Therefore, we could only include 72.5% of medical charts in our analysis due to the lack of physician documentation regarding patients' behavioral changes and their ability to complete activities of daily living. This created a new question as to why physician documentation varied so greatly and whether that could be indicative of the quality of health care that is given in the RGV or whether the comorbid diagnosis of DM led to more frequent follow-ups and better physician documentation [16]. But it is important to note that there is no universal diagnostic test for AD, and therefore, many physicians may not follow a standard process, which may be even more pronounced in a small community-based clinical system [16-19]. For example, a 2010 review noted that from ~1900 articles on AD, over 68 different clinical assessment scales were utilized [19]. We would suggest that future health systems consider following a standardized guideline system and appropriate documentation of activities of daily living to monitor the progression of AD and better understand the needs and support the patient requires.

Study limitations

The main limitation of this study was the small sample size (n = 40), of which only 29 patients could be included. We sought to evaluate a homogeneous group of patients, which may have influenced our findings and introduced selection bias. Future work should evaluate larger databases to extend our observations. Another limitation was the manual grading of AD patients. While we followed published best practices [9], future studies may consider the use of AI to increase reliability and reduce bias. We also acknowledge that our retrospective chart review design may introduce bias, as confirmation of an AD diagnosis through standardized tests or biomarkers could not be fully ascertained, given the wide range of clinical protocols used across the multiple sites from which our data were sourced. However, all enrolled subjects had sufficient information to allow for a GDS score to be tabulated. Thus, there was enough documentation in each chart to ascertain the level of pre-cognitive change or dementia. We encourage future studies to incorporate behavioral assessments, such as the MoCA, to improve diagnostic standardization.

## Conclusions

AD is a multifactorial disease that can be influenced by genetic, environmental, and co-morbid factors. Type 2 DM has been found to have a high co-occurrence with AD, including similar pathophysiology. Hispanic populations, who are at high risk for both AD and DM, may be disproportionately affected by AD and progress faster and/or have a higher grade of AD severity. Here, as a first step, we conducted a pilot study to evaluate the influence of type 2 DM on AD severity in a small sample of Hispanic patients from the Rio Grande Valley. We found that there were no significant differences in AD severity in patients with or without DM. However, our findings highlight that improved healthcare documentation of individuals with AD is required and that a trending relationship does exist between DM and AD severity. Future work should elaborate on our findings with a larger dataset to continue to elucidate the link between DM and AD in Hispanic populations. Identifying the link between the two conditions would be vital to mitigate and slow down disease progression to extend patient QoL.
